# But what do we mean by “health”? A critical perspective on the concept of health in the adolescent transition program of a Norwegian university hospital

**DOI:** 10.1186/s12913-022-08903-5

**Published:** 2022-12-28

**Authors:** Kjersti J. Ø. Fløtten, Isabelle Aujoulat, Vegard B. B. Wyller, Anne Lee Solevåg

**Affiliations:** 1 Department of integrated care and health promotion, Akershus University Hospital, P.O. Box 1000, 1478 Lørenskog, Norway; 2 Department for Paediatric and Adolescent Medicine, Akershus University Hospital, Lørenskog, Norway; 3 Institute for Clinical Medicine, Faculty of Medicine, University of Oslo, Oslo, Norway; 4 UCLouvain, Institute of Health & Society, Brussels, Belgium; 5 Division of Paediatric and Adolescent Medicine, Oslo University Hospital, Oslo, Norway

**Keywords:** Adolescent, Chronic illness, Transition, Patient education, Qualitative research

## Abstract

**Background:**

To understand better what influences the practice of our transition program, we wanted to explore the underlying theory of health.

**Methods:**

We performed a qualitative content analysis of the written material that guides the program, comprising a quality system guideline, two checklists, a guide to health professionals and managers, and three patient brochures.

**Results:**

The analysis resulted in the formulation of three themes; “*Being on top of medical management*”, “*Ability to promote own health*” and “*Awareness of own goals and expectations”.*

**Conclusion:**

Our analysis indicates that the program content revolves mainly around medical management and that other dimensions of health are not emphasised. We question what the goals of the program are and if these goals are explicit and shared among the program stakeholders. An explicit program theory is vital and needs to be evident in material supporting transition programs.

## Background

In 1995, David Rosen pointed out that advances in care for children with a chronic illness or disability had transformed diseases that were previously considered childhood diseases into diseases with childhood onset [1]. He went on to argue that the manner in which we delivered care for adolescents with chronic conditions unfortunately had not kept pace. Fortunately, there has been some development since. Research indicates that poorly planned transfers from paediatric to adult health services can have negative consequences for adolescent patients [2–4] and is experienced by adolescents as challenging [4]. Therefore, a guided educational process starting years before administrative transfer is recommended and has become known as *transition* [3, 5, 6]. Viner argues three things must be in place for safe and effective transition. These are: a cultural shift in staff attitudes and training, an effective transition program, and that adolescents are educated and empowered to truly participate in their own transition [3]. Colver et al identified nine features of transition that they termed *proposed beneficial features* [7]. Furthermore, they found that three features, namely appropriate parent involvement, meeting the adult team and promotion of health self-efficacy, were strongly associated with improved outcomes [8].

The question however remains what an effective transition program is [9, 10]. There is a still ongoing debate in the transition field internationally about how to best evaluate transition programs and what constitutes an effective program [4, 11–13]. In 2015, Suris and Akre performed an international Delphi study of key elements of transition programs and what indicators could be used to evaluate the success of such programs [14]. They found that even though a holistic approach is recommended, consensus elements seemed to be related mainly to health outcomes. In their 2014 review on measurements of transition readiness, Schwartz et al pointed out that the focus has been on age, skills and knowledge, and not social ecological associations [10]. A similar argument is made by South et al in their 2022 comparison of transition readiness instruments [15]. Why does it matter? It matters because; *“it is often maintained that health is one of the major goals of medicine or even*” [16]. However, health is defined in quite different ways, ranging from the biostatistical theory of Boorse [17] to holistic theories such as that of Nordenfelt [18].

The biostatistical theory defines health as the absence of disease. This theory appeals strongly to health professionals; *“as their education and practice extensively is concerned with professional criteria of what falls under DISEASE”* [19]. Carel critiques this theory and argues that it needs to be supplemented because; “*The impact of illness on the ill person remains unaccounted for in this view*” [20]. Moreover, she argues that the biostatistical theory, or as she labels it, naturalistic; “[ …] *theories neglect the loss of agency or incapacity characterising the experience of illness*” [20]. The holistic theory of health; *“[...] primarily aims at giving a positive characterisation of health without leaning on a previous understanding of disease and illness*” [1]. Well-being, ability to perform actions and individual goals are characteristics emphasised in this theory [16, 18].

Nordenfelt argues that the theory of health that is applied, strongly influences how we structure health care [16]. In the context of transition programs, the perceived scope of the program and thus what is seen as key interventions, is likely influenced by an underlying theory of health that defines the problems to be solved and thus the program construction [21]. In other words, by using a biostatistical theory of health, we risk having health care with a more narrow focus than what is beneficial for the adolescents. In the review of Sattoe et al on self-management interventions for adolescents with chronic conditions, 67.9% of the included publications did not define the theoretical basis of the interventions [12]. According to Bickman; *“Every program has a theory. Unfortunately, it may be implicit, fragmented, and not well conceptualized”* [21]. This lack of clarity may result in different understandings of program goals by different program stakeholders. Without an explicit and shared understanding, the stakeholders could seemingly be working towards the same goals while in fact they are not. Furthermore, Bickman argues that: “*Moreover, developers and implementers often do not have in-depth training in theories of behavior. These factors all contribute to the too-common fact that programs lack explicit theory or that the theory espoused may be implausible*” [21]. The program theory influences all three components highlighted by Viner as important for successful transition, e.g., definitions of an effective program, and what areas of empowerment are being addressed.

When the transition program at Akershus University Hospital (Ahus) was developed from 2012 to 2016, it was moulded from existing international programs [22]. Similar to many of these programs, we developed written material to frame the program [13, 23–26]. The first author was involved in initial translations of material from English. These documents should inform adolescents about transition, but also serve as tools for health professionals. As part of the continuous work on improving our program, we initiated an action research project (HI-FIVE). We chose action research as an approach because it not only provided an opportunity to evaluate the program, but also allows for program development through action and continuous learning [27, 28]. Our initial intentions were to monitor the program implementation and to evaluate whether the program supported empowerment. We framed our project with the five steps of action research described by Kemmis et al, and started by investigating how the program came to be, how is it practiced and what consequences this has for the program content [29]. Using international literature as a point of departure, we considered it appropriate in the first step of this process to study the dimensions of health that are emphasised in our program, how this came to be and whether it has untoward consequences [29]. Before we could evaluate the effectiveness of our program and make suggestions for improvement, we needed to know how the implementation was actually proceeding.

The first step of HI-FIVE resulted in three successive modes of data generation that provided a basis for discussions in step two about where to go from here. These were; 1) a content analysis of the program material, 2) key informant interviews with program developers and 3) interviews with physicians and nurses that are currently responsible for the day-to-day running of the program. In this article, we report on the analysis of the program material. According to Charmaz; *“Documents represents discourses and accounts. As a discourse, a document follows certain conventions and assumes embedded meanings”* [30]. To paraphrase Prior, the written material of the program should not be seen merely as containers of words but as actors that influence the social interaction in the program [31]. Thus, analysis of their content is highly relevant. We approached the material with the following questions:

1. What can we learn about the concept of health underlying our program through analysis of the program material?
2. What consequences do we foresee these findings to potentially have for the program?

## Methods

### Qualitative content analysis of the written material

The written program material expresses opinions and ideas about the program [32] and is an information source for health professionals, adolescents and their parents/guardians about the program and transition in general. Charmaz states that; *“A study of what a document does can include the following: 1) what its originators intended to accomplish; 2) the process of producing the document; 3) what and whom the document affects; 4) how various audiences interpret it; and 5) how, when, and to what extent these audiences use the document.”* [30]. The researcher has a unique role in the interpretation of the text because they are reading the text not as a member of the main target group, but with the intention of understanding the views and opinions of its originators [32]. None of the strategies of interpretation are mutually exclusive or strictly separated, but provides the researcher with tools to reflect on their choice of main focus [32]. We acknowledge that a text can have multiple meanings and that our analysis is one story [33].

The aim of the analysis was to readdress our understanding of the program we are running. Our idea was that analysing what concept of health is used in the program would give us an opportunity to discuss the value basis of the program, or put differently, the normative program theory [34]. According to Chen, this part of a program theory is often taken for granted and rarely explicitly expressed [34]. Bickman states; *“A program theory should also be able to clarify the relationship between the program and the problem. A program is typically designed to solve some problem for a targeted group. It is assumed that if the program is implemented properly it will affect the problem afflicting the group”* [21]. Through investigating how the documents address the content of the program, we could get an idea about what the developers perceive as vital for the development and maintenance of *health*. We aimed to interpret what concept(s) of health the authors wanted to convey, as a basis for discussions of whether this value base is desirable [29]. Charmaz argue that: “*Written texts not only serves as records, but also explore, explain, justify, and/or foretell action [ …*]” [30]. If the written material is to serve as frames for action in the program, we should reflect on the appropriateness of the frames.

### The written material

Seven items make up the material and constitute the *units of analysis* [35]. These items are; a hospital quality system guideline, two checklists; *My Health* and *Ready for transfer* [36, 37], a guide to health professionals and managers [38] and lastly, three brochures; *My Rights*, *My General Practitioner* and *Operation independence* [39–41]. They are all applicable across diagnoses [22]. All items are first editions and were developed by translating material developed internationally and adapting them to the Norwegian context with input from the hospital Youth Council [22]. A general idea was that the material should be easily accessible and written in an inclusive language. For usability in a hectic hospital environment, a brief brochure format was favoured, and overall, the entire material (excluding cover pages or full-page pictures) comprises of 20 pages of highly condensed text.

### The analysis

We performed a qualitative content analysis of the material as outlined by Graneheim and Lundman [35]. We approached the texts inductively i.e., without a predefined grid or model. Qualitative content analysis provides flexibility because it focuses on both manifest (what the text says) and latent content (what the text talks about) [35]. In qualitative content analysis one looks for words, sentences or paragraphs that convey a meaning relevant to the research question [32], also referred to as *meaning units* [35]. In all cases but the guideline, analysis was performed on the English translations of the material.

The first author performed the preliminary analysis. The process started by reading the whole text multiple times to get a sense of what it says. Then the text was read with our research questions in mind, and meaning units were highlighted by hand with a text marker. The meaning units were then entered into an analysis grid that outlines the steps of qualitative content analysis (Table 1).

**Table 1:** Outline of analysis grid

Units of analysis: Written material transition program	Manifest content				Latent content
Meaning unit	Condensed meaning unit	Code	Sub category	Category	Theme

The next step was condensation, meaning that the text was shortened where possible, but the core was still preserved [35]. Parts of the guideline and the checklists were already highly condensed and further condensation was not necessary nor desirable [42]. After condensation, the shortened meaning units were labelled by creating codes that were still close to the original text [42]. The analysis grids of each item were then distributed to two of the co-authors (ALS and IA) for discussion. Graneheim and colleagues stress that; “*Even if these descriptions point to a linear process, it is important to bear in mind that the process of analysis involves a back and forth movement between the whole and parts of the text*” [35]. For us, this entailed a back and forth movement between the text as a whole and the meaning units to secure that the meaning units preserved the material context and meaning [42]. When labelling with codes, we were also constantly revisiting the meaning units for possible further division [42]. Having condensed text as our point of departure, several meaning units could be identified within short passages of text.

The coding was followed by development of categories across all seven units of analysis [35]. “*As we see it, a category refers mainly to a descriptive level of content and can thus be seen as an expression of the manifest content of the text*” [35]. Categories are still close to the original text with a low level of abstraction. Themes were then formulated. Themes are abstract and recurring [43, 44] and answer the question: “*What is this expression an example of*” [45].

## Results

The analysis resulted in the formulation of three themes; *Being on top of medical management*, *Ability to promote own health* and *Awareness of own goals and expectations* (Table 2)*.* Meaning units were drawn from all items. We found no explicit definition of health in the material.

**Table 2:** Table of codes, subcategories, categories and themes

Theme	Being on top of disease management				Ability to promote own health		Awareness of own goals and expectations	
**Cate-gory**	Medical management				Promoting own health		Goals	Expectations
**Sub cate-gory**	Knowledge of medical condition and treatment	Responsibility for disease and treatment	Skills to master medical condition	Manoeuvring health care system	Responsibility for own health	Knowledge to promote own health		
**Code**	Knowledge of body, diagnose and treat-ment Knowing medications Knowing medical procedures Knowing equipment Knowing when to seek help Knowing impact of medical condition	Gaining respon-sibility for treatment Taking respon-sibility for medication Taking respon-sibility for treatment Complying to treatment plan	Gaining experience to handle medical aspects	Manoeuvring mental health services Knowledge and skills for adult services	Increased responsibility for own health Promoting and maintaining good health Taking care of own health	Knowing benefits of lifestyle choices Being aware of own health	Becoming aware of own goals	Idea of normality clashes with demands Living life like “everyone else” Questioning life with a disease Denial of disease Becoming aware of own expectations

Text sections not deemed relevant to constitute meaning units in our analysis included instructions on responsibilities and descriptions of roles addressed to different health professionals in the quality system guideline. We also excluded text specifying health care rights, describing technicalities of the health care system, explaining the role of general practitioners, or the difference between paediatric and adult health service. These text excerpts were excluded because they provided systems knowledge and not information about the definition of health, i.e., they did not contain information related to our research question.

The result of our analysis is presented by theme and supported with examples drawn from the text.

### Being on top of disease management

This theme constituted one category, namely *medical management.* The majority of meaning units were grouped under this theme. They were characterised by a focus on the adolescent receiving information, building knowledge, gaining ability to take care of and responsibility for own *disease*, *treatment*, *medication* or *health challenge*. Examples of this are the following excerpts:

*“I understand the medical terms that pertain to my health condition or disability”* [37].

*“Adolescents require realistic knowledge about their health challenge and different situations that may appear”* [38].

It is argued that having this knowledge will support the adolescent, increasing their ability to manoeuvre the adult health services, living a *normal* life and managing own health:

*“Having good knowledge of one’s own body, diagnose and treatment provides the best possible circumstances for being able to live one’s life like “everybody else” during adolescence”* [38].

*“I know who to contact if I experience mental problems”* [37].

A disease-oriented and medical focus is maintained in all meaning units belonging to this theme.

### Ability to promote own health

One category belonged to this theme. Meaning units in this theme also focus on increased responsibility and gaining knowledge, but are phrased with regards to *own health* and not *disease*.

*“Transition is a process where adolescents with long-term health problems receive individually adapted information and increased responsibility for their own health”* [38].

*“A good first encounter with the adult clinic will provide a basis for developing a new trusting relationship and continued good cooperation in order to promote and maintain a good health condition”* [38].

Promotion of health is also portrayed as concerning knowledge about lifestyle choices such as nutrition, alcohol and exercise. An example drawn from the checklists is:

*“I know the benefits of taking care of myself properly and how nutrition, sleep habits and stress affect me”* [36].

The only time the phrase *health promotion* occurs, is concerning consultations between health professionals and adolescents:

*“Health care personnel must have the knowledge, skills and attitude that enable good, health-promoting encounters with adolescents.”* [38]. Details on what constitutes a health-promoting encounter is not provided.

### Awareness of own goals and expectations

This theme had two categories, namely *goals* and *expectations.* The meaning units are short excepts from the guide to health care professionals and managers about denial of disease in adolescence and that there might be times when adolescents question what life will be like living with a disease. There are also meaning units that convey that adolescents strive to be *like everyone else* and *normal*.

*“They are often times so controlled by the idea of normality and to “be like everybody else” that it clashes completely with the demands of following a treatment plan”* [38].

Lastly, there is the topic of the need for adolescents, parents and carers to become aware of the adolescents’ own goals and expectations in relation to their disease and treatment.

*“Professionals, parents and adolescents may have completely different goals for mastering the health challenge”* [38].

*“Through dialogue and reflection, the adolescent is independently able to become aware of his or her goals and expectations, as are their parents and professionals”* [38].

Table 2 outlines themes, categories, sub-categories and codes.

## Discussion

Our analysis indicates that *Being on top of medical management* is at the forefront in our program too with the majority of text categorised to this theme. It is interesting that a large proportion of the meaning units drawn from the material geared to adolescents themselves, such as the checklists, were grouped under this theme. This echoes findings in literature reviews such as Sattoe et al., Morsa et al. Schwartz et al. and South et al. [10, 12, 15, 46] that support Suris and Akre’s findings regarding transition interventions internationally giving less attention to psychosocial dimensions of health [12, 46]. Given that our program was moulded from international programs, this result might not be surprising. The question however, remains if this is a well thought through strategic choice or an unintended mishap due to underlying assumptions not being discussed [21]. What discussions are had when one takes an interest in programs created internationally?

It can be argued that the themes *Ability to promote own health* and *Awareness of own goals and expectations* touches topics that focus less on medical management. We also acknowledge that amount of text does not automatically equal importance. However, as can be seen in the quotes, how the topics of *goals* and *expectations* are presented in the material is largely linked to being able to follow a treatment plan or to *mastering the health challenge.* The meaning units in the themes *Being on top of medical management* and *Ability to manage own health* were sometimes interchangeable. The two themes may in fact have stayed separate due to language rather than content. The units that were allotted to medical management very clearly related to *disease*, *treatment*, *health challenge* and *handling medical aspects*. In other instances, we could not differentiate whether the use of the term *health* referred to health in a narrow or wide sense. An example is the following excerpt: *“Acknowledge wishes to take responsibility for one’s own health when such are expressed”* [38]. The highly condensed text in the material did not provide rich details. We thus found it appropriate to keep the two themes separate.

In our opinion, the format of the material provides a challenge. Yes, it is short, and thus possibly more easily accessible. However, it leaves opportunities for differences in interpretations of goals. This, in our opinion, is not a challenge in the transition field alone, but is part of the larger debate about the format of patient information in general. For instance, the phrase “*health promoting encounter*” is used, but no description of what a health promoting encounter entails is provided [38]. Since there is no explicit definition in the material, identifying examples of health promotion requires prior knowledge about what it is. The material focuses mainly on health behaviour rather than on the meaning-making aspects of health. It does not explicitly include more existential parts of health or phenomenological perspectives such as argued by Carel [20].

We can of course not conclude from this analysis alone that these topics are not raised in the program at all. A program is more than written material. Nevertheless, the material has a central position in the program, as a guide to health professionals, adolescents and parents. We question what kind of frame it provides. In 2020, The Norwegian Association of Youth with Disabilities performed a survey among 253 adolescents with disabilities or chronic illness [47] and found that adolescents want to talk about more than their diagnosis and that they find it difficult to initiate such a conversation. Fifty percent of the respondents stated that health professionals do not address topics such as thoughts, feelings or self-image. Thus, it seems vital that health professionals initiate conversations on these dimensions of health. We do not doubt that many health professionals do raise these kinds of questions with their patients. However, judging from the adolescents’ feedback in the mentioned survey, not nearly many enough. A goal of introducing a program would be that it provides some kind of frame of reference for and unity in the services provided. Betz et al conclude in their 2013 systematic review on adolescents’ perspectives on health care transition that the focus in studies has been on transfer of care and state that few studies have focused on the support needed to acquire the perceived relevant knowledge and skills [48]. That is the exact gap this document analysis points out too.

The stated aims of the program are to strengthen the adolescents’ knowledge about their own health, and empower them to take responsibility for their own health and treatment [22]. The short- and long-term goals of the program include improved quality of services and health promotion. As such, it can be argued that the program aims for increased ability to manage own health. The analysis of the material does however make us question what is meant by *health* and if the focus is broad enough. It also leaves us with the question of whether the focus is too much on knowledge *provision* rather than knowledge *co-creation.* When we take what we find in our own material and put it into the wider context of the debate internationally, with arguments made by the earlier referenced reviews of a too narrow focus, we question whether we are the only program where this is an issue. In fact, Farre and McDonagh argue that it is time to get the focus back on the life transitions of the adolescents rather than the structural boundaries between services [49]. One may speculate whether the transition field has in fact lost itself in the frequently referenced definition of transition as *the purposeful planned movement* of the adolescents rather than sticking to a wider definition of transition argued by for instance Meleis et al [50].

Another relevant way of approaching the question of theoretical basis would be to ask whether transition programs are traditional patient education programs or self-management education programs [51]. Traditional patient education focuses on technical skills and differs from self-management education [51]. At the core of this difference is the underlying theory of what health is and theories of how people learn, in other words, what the normative theory of a program is [34]. Traditional patient education has a narrower, biomedical definition of health combined with a learning theory that disease-specific knowledge creates behavioural change [51]. Self-management education on the other hand, has a goal of increased self-efficacy through learning problem-solving skills [51] and a conviction that increased self-efficacy strengthens productive self-management. It all rests on the patient’s own formulation of problems [52]. To return to our material as an example, even though it touches on topics such as formulation of own goals, the goals are defined in relation to disease management. Thus, our transition program seems to have a more traditional approach to patient education. Knowing how we moulded our program with inspiration from programs internationally, the question of traditional patient education versus self-management education seems to be a timely question to ask not only about our program but also about transition programs in general. Is it taken for granted that knowledge about own disease and skills in managing it automatically creates behaviour change and better coping?

It is, as earlier mentioned, stated in our material that empowerment is a goal. However, according to Aujoulat et al [53], empowerment is often defined as a process focused on behavioural change, where health professionals focus on supporting the patients in developing knowledge and taking control over their body, illness and treatment. Defined in such a way one could argue that gaining the knowledge and abilities referred to in the material would empower the adolescents. Still, Aujoulat et al contend that identity should be addressed as part of empowerment [53]. In other words more aspects of identity- and emotional management should be brought up [54].

According to Carel it is important for the chronically ill that health is defined as something more than the absence of disease [20]. It is important that health be defined as something more than taking your medication and complying with doctor’s orders. Furthermore, Paterson argues that the Shifting Perspectives Model indicates a need to acknowledge the individuals shifting perspectives of their illness [55]. Corbin and Strauss argue that the continuous tasks of living with a chronic illness can be divided into three dimensions; medical management, identity or role management, and emotional management [51, 52]. These are lifelong tasks and have implications for how we should build a transition program [52]. Similarly, Audulv et al write about the internal negotiation the individual has concerning self-management [56], a process that frequently sets different goals up against each other such as medical vs social goals. According to Audulv et al this whole process starts with the individuals definition of health [56]. A program that addresses only one of these dimensions will thus be inadequate.

Issues that are raised in the material could to a large extent be classified as actions to increase adolescent health literacy, but are not explicitly named as such. This is not surprising as the explicit mention of health literacy as a goal of transition programs is quite infrequent in the international literature on transition. Much of the material does however concern “*knowledge, motivation and competency to access, understand, appraise and apply health information*” [57]. In fact, parts of the text we excluded focuses on health literacy, such as knowledge about the health care system and health care rights.

## Conclusion

The analysis of the written program material indicates that we echo international programs in a focus primarily on medical management. The question however remains if this was a deliberate choice and what impact this focus has on health professionals’ practice. Hoffmann [19] argues that health professionals use more than one model of health at the same time and that this may not necessarily constitute a problem. The problem arises if and when there is no awareness of this and no explicit choice is made.

### Practical implications

Our study contributes to awareness of the need for critical reflections on the normative theories that guide transition programs as well as patient education programs in general. If we in our eager to develop programs do not reflect on the underlying program theories, we may risk creating programs with an unintended narrow focus. This should be considered when developing and evaluating transition programs internationally. For us specifically, the results had implications for the process of our action research project and thus for development of the program. We have explored the topic further in the aforementioned key informant interviews and interviews with health professionals. Results from all these processes of data generation form a base for discussions about the practice of the program. This may imply changes to the program material, but just as important a need to establish a common understanding of the program. The ultimate goal being to strengthen our services for adolescent patients.

## Data Availability

The datasets generated during and/or analysed during the current study are available from the corresponding author on reasonable request.

## Abbreviations

Ahus: Akershus University Hosptial
HI-FIVE: Health LIteracy For young Individuals, its Value for Empowerment and successful transition – working title of main project
